# When Diarrhea Tells a Deeper Story: A Curious Case of Metastatic Medullary Thyroid Carcinoma

**DOI:** 10.7759/cureus.86542

**Published:** 2025-06-22

**Authors:** Milan Shrestha, Ajay K Yadav, Ramila Shrestha, Dibas Khadka, Mukesh S Paudel

**Affiliations:** 1 Department of Gastroenterology, Bir Hospital, National Academy of Medical Sciences, Kathmandu, NPL

**Keywords:** calcitonin, carcinoembryonic antigen, chronic diarrhea, medullary thyroid carcinoma, metastatic, multiple endocrine neoplasia type 2, palpable neck mass, sporadic medullary thyroid carcinoma

## Abstract

Chronic diarrhea is characterized by an increased frequency of loose stools persisting for more than four weeks. Diagnosing chronic diarrhea requires a comprehensive evaluation that includes a detailed medical history, physical examination, and investigations guided by diagnostic clues. Diarrhea is often the presenting symptom of various underlying conditions. Medullary thyroid carcinoma is a rare neuroendocrine tumor derived from the parafollicular C cells of the thyroid gland. It can occur sporadically or be inherited as a part of a syndrome, such as multiple endocrine neoplasia type 2. Chronic diarrhea can be a presenting symptom of medullary thyroid carcinoma and should always be considered in the differential diagnosis, particularly if the patient has a palpable thyroid nodule. This case report describes a 65-year-old woman with a rare metastatic medullary thyroid carcinoma who presented with chronic diarrhea and a palpable thyroid mass. Fine needle aspiration cytology and histopathological examination of the thyroid gland confirmed a diagnosis of medullary thyroid carcinoma. The diagnosis was also supported by increased calcitonin and carcinoembryonic antigen levels, which can be used for prognostication and monitoring response to therapy. She was treated with a tyrosine kinase inhibitor, which substantially alleviated her diarrhea, but she succumbed eventually to metastatic complications.

## Introduction

Medullary thyroid carcinoma (MTC) is a rare type of thyroid cancer accounting for 1-2% of all thyroid malignancies. These tumors develop from the parafollicular C cells of the thyroid gland. Typically, these tumors cause painless swelling in the neck along with painless enlargement of cervical lymph nodes. Clinical symptoms are usually related to local invasion and hormone production. Hormone-related symptoms such as flushing and diarrhea are often associated with advanced stages of the disease [1,2]. The case being discussed here involves metastatic MTC presenting with chronic diarrhea.

## Case presentation

**Figure 1:**
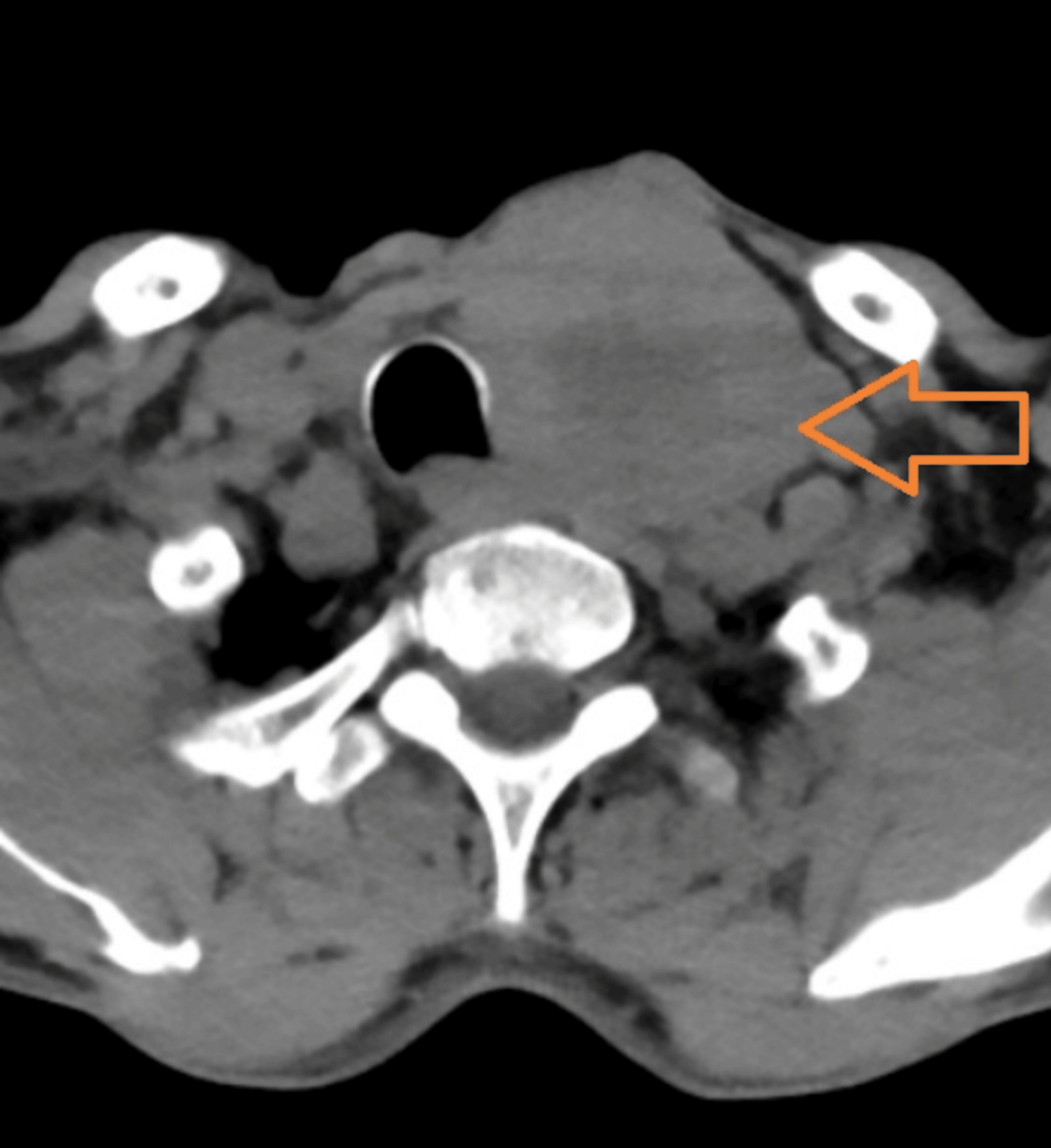
Contrast-enhanced computed tomography of the neck showing a malignant mass in the left thyroid gland measuring 31 x 27 x 26 mm (orange arrow).

A 65-year-old lady from central Nepal with no comorbidities presented with a history of loose stools and intermittent abdominal discomfort for the last six months. She reported experiencing 15-20 episodes of watery, large-volume, non-mucoid stool without blood, along with intermittent crampy abdominal pain before defecation. Diarrhea had no specific association with meals. She had not been taking any medications, including complementary and alternative medications. These episodes were not associated with diurnal variation. The patient also had hoarseness of voice and painless, progressively enlarging swelling in the neck for the last four months. She had a painless, solid mass over the left side of the neck, which was non-tender, approximately 2 × 2 cm in size, without skin changes. The swelling moved with swallowing, as well as with protrusion of the tongue, and was mobile. She also had palpable left cervical group of lymph nodes, which were hard, smooth, painless, and non-matted. The patient had a history of involuntary weight loss of 20 kg over six months. Initial stool workup was negative for fecal leukocytes, ova/parasite, and *Clostridioides difficile* infection. Stool osmolar gap was 10 mosmol/kg, suggestive of secretory diarrhea. Complete blood counts, thyroid function tests, renal and liver function tests, and C-reactive protein (CRP) were within normal limits. Esophagogastroduodenoscopy (EGD) showed normal gastric and duodenal mucosa. Colonoscopy was unremarkable for any pathology, both endoscopically and histologically. Ultrasonography (USG) of the neck reported a neck mass with cervical lymphadenopathy. Contrast-enhanced computed tomography (CECT) of the neck, chest, abdomen, and pelvis revealed a malignant mass (31 x 27 x 26 mm) in the left thyroid gland with cervical lymphadenopathy, periportal, and retroperitoneal lymphadenopathy (Figure 1). It also indicated an enhancing mass in the liver and multiple vertebrae, a bulky adrenal gland, anterior mediastinal nodules, and multiple nodules in bilateral lungs with mild ascites, all suggesting metastasis.

**Figure 2:**
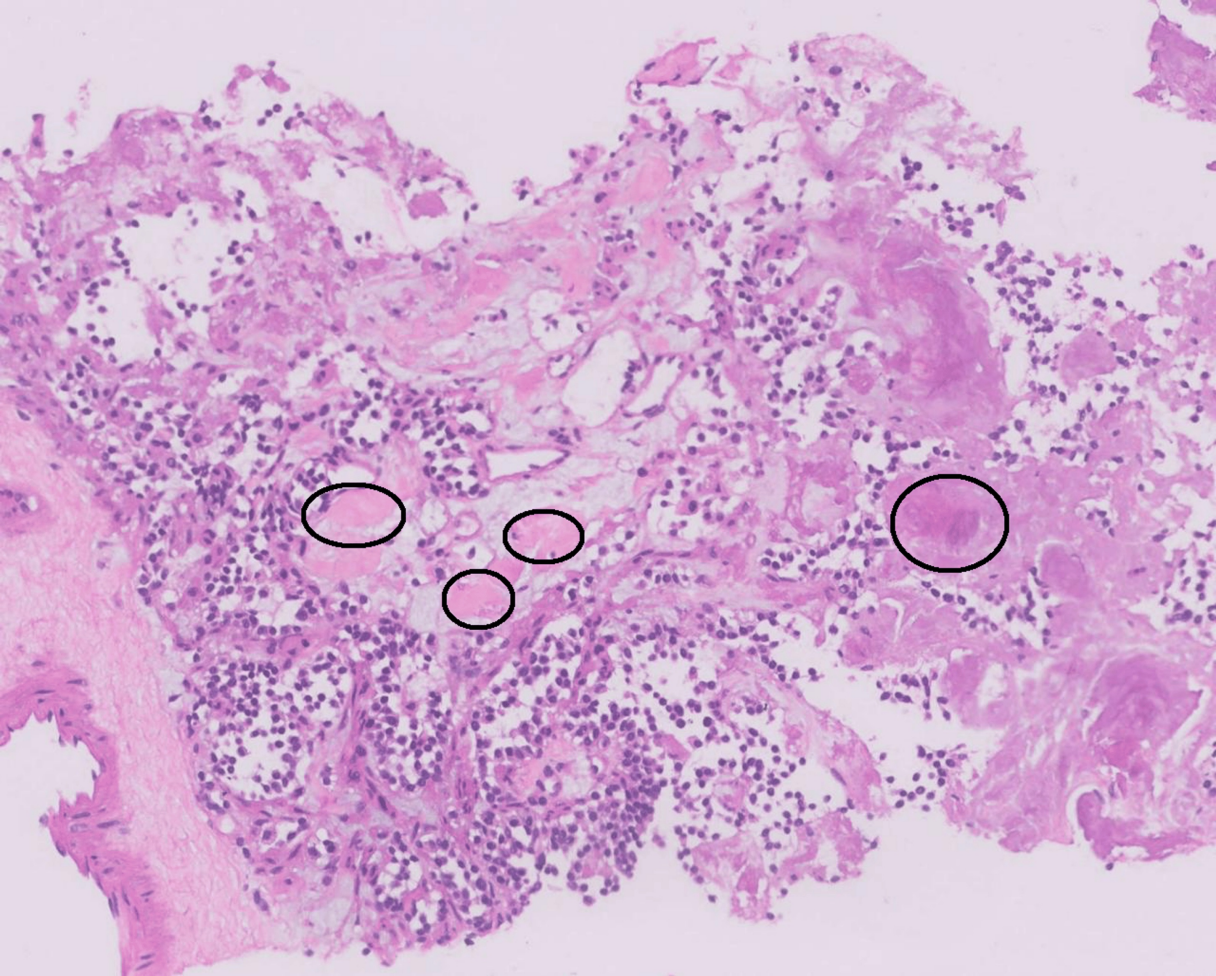
Histopathology of hematoxylin and eosin-stained thyroid sections shows round cells with indistinct nuclei, coarse chromatin, scant to moderate amphophilic cytoplasm, and amorphous eosinophilic amyloid deposits in the stroma (black circles).

**Figure 3:**
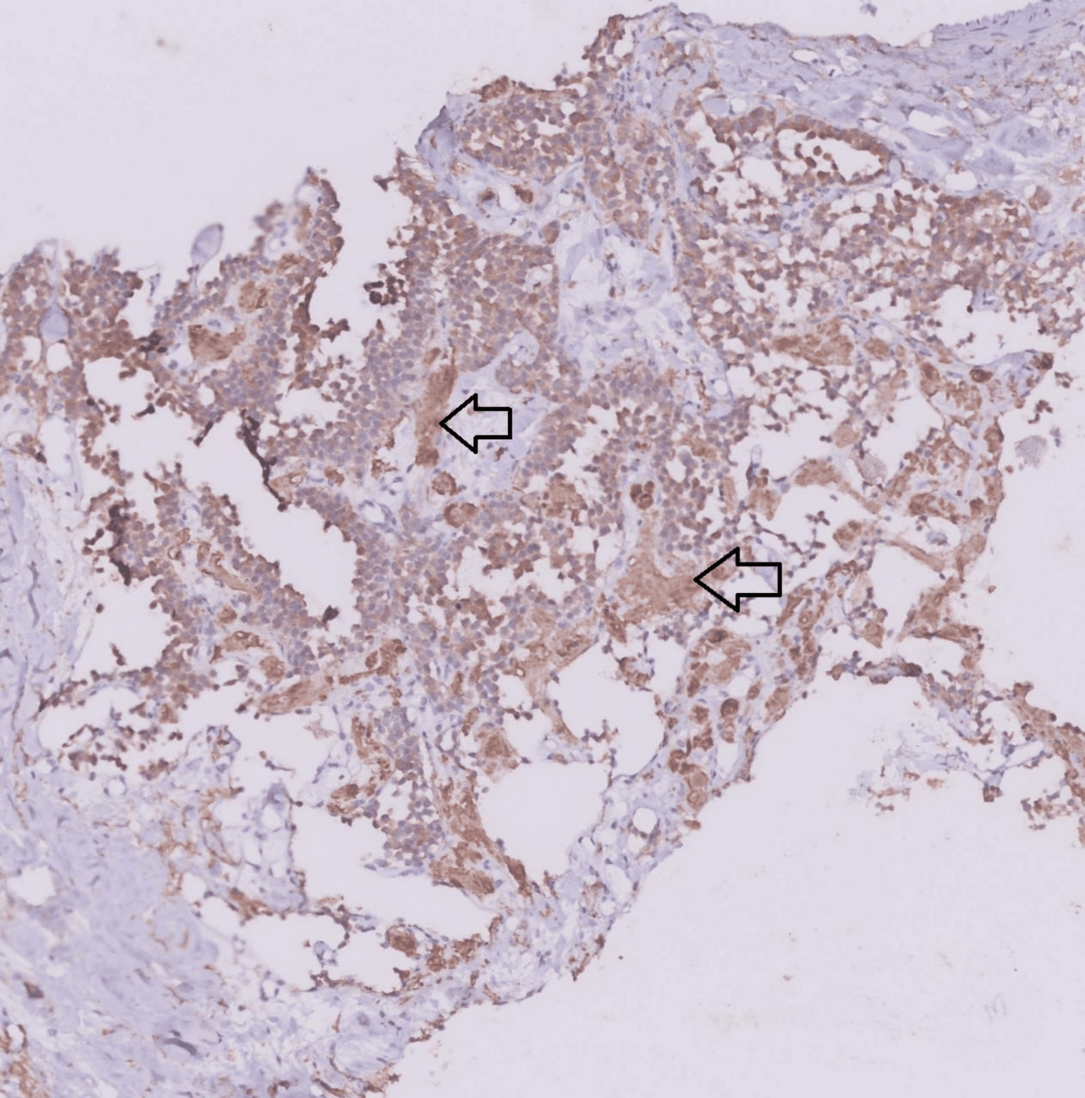
Immunohistochemistry shows tumor cell positivity for calcitonin (black arrows).

USG-guided fine needle biopsy (FNB) of the thyroid mass revealed round cells with indistinct nuclei, coarse chromatin, scant to moderate amphophilic cytoplasm, and amorphous eosinophilic amyloid deposits in the stroma, which were suggestive of MTC (Figure 2). Serum calcitonin > 2000 pg/ml (normal value < 11.50 pg/ml) and carcinoembryonic antigen (CEA) of 3138 ng/ml (normal value < 2.5 pg/ml) were significantly higher. Intact parathormone (PTH) and urinary vanillylmandelic acid (VMA) were within normal limits. In an immunohistochemistry (IHC) study, the tumor stained positive for calcitonin, cytokeratin 7 (CK7), thyroid transcription factor 1 (TTF1), and synaptophysin with an intermediate Kiel 67 (Ki-67) index (Figure 3). Rearranged during transfection (RET) proto-oncogene fluorescent in situ hybridization (FISH) analysis revealed a negative status, concluding a sporadic MTC.

The patient was initially treated with loperamide and octreotide; however, these interventions did not yield significant improvement in her symptoms. Following an oncology consultation, the patient was prescribed lenvatinib, a tyrosine kinase inhibitor, which substantially alleviated her diarrhea. This was accompanied by a noteworthy reduction in pretreatment calcitonin and CEA levels, but did not reach normal values. Nevertheless, despite this positive response, the patient ultimately succumbed to metastatic disease six months later due to respiratory failure.

## Discussion

MTC, a rare neuroendocrine tumor arising from parafollicular C cells of the thyroid gland, represents 1-2% of thyroid malignancies. It exhibits no significant gender or ethnicity differences. Approximately 80% of these cases occur sporadically, with the remaining 20% being hereditary and associated with the RET mutation. Sporadic MTC typically appears in individuals aged 50 to 60 years, similar to this case, while hereditary cases usually present in the second and third decades of life [1,2]. Patients with MTC usually have a painless mass in the upper neck area. Approximately 70% of patients exhibit cervical lymphadenopathy. At the diagnosis, around 13% of cases are metastatic, often with high levels of calcitonin (above 150 pg/mL) [2]. Common sites of metastasis include the liver (49-62%), lungs and mediastinum (33-35%), and bone (40-74%) [3]. Our patient also presented with a painless thyroid mass along with metastasis to the liver, lungs, vertebrae, and mediastinum.

Nodal positivity, vascular invasion, extrathyroidal extension, high pathological grade, presence of metastatic disease, and age over 55 years have all been shown to predict shorter survival [4]. All of these observations were evident in our case, and tragically, she lived just a year after her initial presentation. Rarely, tumoral secretion of various molecular factors, such as calcitonin, can cause diarrhea in approximately 30% of patients, particularly in advanced or metastatic disease. High plasma calcitonin levels increase electrolyte secretion via mediators such as prostaglandins, serotonin, or vasoactive intestinal peptide (VIP). However, recent studies on pathophysiologic mechanisms of diarrhea in MTC found normal levels of these mediators, normal small bowel absorption, and normal gastric and small bowel transit. Markedly decreased colon water absorption and colonic transit time were the major factors for diarrhea [5].

Fine needle aspiration (FNA) or biopsy (FNB), preferably under USG guidance, is essential for determining malignancy in a thyroid nodule [6]. The most important cytologic criteria with MTC's FNA/FNB are a dispersed cell pattern of polygonal or triangular cells, azurophilic cytoplasmic granules, and extremely eccentrically placed nuclei with coarsely granular chromatin and amyloid [7]. High calcitonin can be used in conjunction with FNA/FNB for the diagnosis. Positive predictive values of MTC rise with higher titers of calcitonin [8]. Preoperative CEA levels correlate with tumor size. These markers are also useful during the follow-up of patients undergoing surgical resection [9].

Histopathological analysis is the gold standard with positive immunohistochemical expression for calcitonin, synaptophysin, chromogranin, and CEA. Nearly all patients with hereditary forms have germline mutations in the gene encoding the RET proto-oncogene. Somatic RET mutations are found in up to 65% of patients with sporadic MTCs. Around 50-60% of cases with germline RET mutations, particularly the M918 T mutation, are associated with an aggressive clinical outcome. For patients with a RET oncogene mutation, it is advisable to screen for hyperparathyroidism and pheochromocytoma to rule out multiple endocrine neoplasia type 2 (MEN-2) syndrome [9].

For locally advanced and metastatic MTC, chemotherapy and radiation have been largely ineffective. Tyrosine kinase inhibitors (TKI) with selective activity against RET, vascular endothelial growth factor receptor (VEGFR), and epidermal growth factor receptor (EGFR) are indicated for such tumors. The FDA has approved pralsetinib and selpercatinib for patients with a RET oncogene mutation [10]. This patient was treated with lenvatinib, which led to substantial alleviation of her diarrhea, but unfortunately, she succumbed to metastatic complications.

## Conclusions

MTC is a rare thyroid malignancy. Chronic diarrhea can be a presenting symptom in advanced and metastatic disease, which also confers a poor prognosis in patients with MTC. It should be considered in the differential diagnosis of chronic diarrhea, particularly if the patient has a palpable thyroid nodule. Given the rarity of the disease and its atypical presentation, we are reporting this case to contribute to the existing sparse literature on MTC and chronic diarrhea.
